# How to Find the Upper Extremities of the Divided Tendons in Cases of Division of the Flexor Tendon of the Fingers

**Published:** 1894-01

**Authors:** 


### HOW TO FIND THE UPPER EXTREMITIES OF THE DIVIDED TENDONS IN CASES OF DIVISION OF THE FLEXOR TENDONS OF THE FINGERS.

At a meeting of the Surgical Society, Dr. Felizet said: "I have observed a tolerably large number of cases of division of the flexor tendons of the fingers in which I had recourse, with success, to a very simple procedure in order to facilitate the search for the upper extremities of the divided tendons. My observations do not apply to wounds of the tendons of the thumb, but only to those involving the flexor tendons of the four fingers, whether in the hand or at the wrist.

"The method of procedure I employ to trace the upper end of the divided tendon is based on the anatomical disposition of the fine fabrous bands which bind the tendons together. For example, to find the upper extremity of the flexor tendon of the middle finger, the other fingers, or at any rate the ring finger, should be fully extended, and as a result the cut end of the tendon looked for is made to emerge, being dragged upon by the fibrous bands which bind it to the other tendons.

"The same principle can be applied in suturing a divided tendon. The neighboring fingers, the tendons of which are still entire, being fully extended, the cut extremities of the injured tendon approach each other, and the suture is rendered more secure by the upper end being stiched to one of the adjoining tendons before the divided extremities are united."—*Medical Week.*

